# Basophil activation in insect venom allergy: comparison of an established test using liquid reagents with a test using 5-color tubes with dried antibody reagents

**DOI:** 10.1186/s12865-024-00616-0

**Published:** 2024-04-27

**Authors:** Sebastian Waldherr, Miriam Hils, Martin Köberle, Knut Brockow, Ulf Darsow, Simon Blank, Tilo Biedermann, Bernadette Eberlein

**Affiliations:** 1 Department of Dermatology and Allergy Biederstein, School of Medicine and Health, Technical University of Munich, Munich, Germany; 2 Center of Allergy and Environment (ZAUM), School of Medicine and Health & Helmholtz Center Munich, Technical University of Munich, German Research Center for Environmental Health, Munich, Germany

**Keywords:** Basophil activation test, Liquid reagents, Dried antibody reagents, Insect venom allergy

## Abstract

**Background:**

Flow cytometry-based basophil activation tests (BAT) have been performed with various modifications, differing in the use of distinct identification and activation markers. Established tests use liquid reagents while a new development involves the use of tubes with dried antibody reagents. The aim of this pilot study was to compare these two techniques in patients with insect venom allergy.

**Methods:**

Seventeen patients with an insect venom allergy were included in the study. The established “BAT 1” utilizes conventional antibody solutions of anti-CCR3 for basophil identification and anti-CD63 to assess basophil activation, whereas “BAT 2” uses dried anti-CD45, anti-CD3, anti-CRTH2, anti-203c and anti-CD63 for identification and activation measurement of basophils. Negative and positive controls as well as incubations with honey bee venom and yellow jacket venom at three concentrations were performed.

**Results:**

Seven patients had to be excluded due to low basophil counts, high values in negative controls or negative positive controls. For the remaining 10 patients the overall mean (± SD) difference in activated basophils between the two tests was 0.2 (± 12.2) %P. In a Bland-Altman plot, the limit of agreement (LoA) ranged from 24.0 to -23.7. In the qualitative evaluation (value below/above cut-off) Cohen’s kappa was 0.77 indicating substantial agreement. BAT 2 took longer to perform than BAT 1 and was more expensive.

**Conclusion:**

The BAT 2 technique represents an interesting innovation, however, it was found to be less suitable compared to an established BAT for the routine diagnosis of insect venom allergies.

**Supplementary Information:**

The online version contains supplementary material available at 10.1186/s12865-024-00616-0.

## Introduction

The basophil activation test (BAT) is an ex vivo provocation assay based on allergen-induced activation of basophils that allows for the in vitro quantification of activated basophils by flow cytometry. The test is widely used for advanced allergy diagnostics and was developed for type-I-allergy diagnostics by Sainte-Laudy et al. in the mid-1990s after the discovery of CD63 as an activation marker of basophils by Knol et al., which is now most often used for this purpose [10, 16].

Since the introduction of the BAT as a commercially available test at the beginning of the 2000s [18] it has been performed in various modifications, differing in the use of various identification and activation markers.

Until the year 2008, mainly anti-IgE protocols were used [6], but other basophil identification strategies using anti-CD123^high^/HLA-DR^neg^ and anti-CRTH2^high^/anti-CD3^neg^ were also published [4, 8]. Anti-CD203c can be used as an identification as well as an activation marker [5]. Anti-CCR3 is another identification marker, which has been shown to be more robust than anti-CD123^high^/anti-HLA-DR^neg^ and anti-IgE [9, 14]. Besides CD63, CD203c is widely accepted as an activation marker leading to slightly better sensitivity but at the expense of specificity for example in insect venom allergy [7].

Recently, the BAT evolved from the use of manually analyzed single tubes to automated analysis of 96-well plates to process many samples [1]. A new technology provides tubes that contain a dry antibody panel coating adhered to the bottom of the tube. It enables pipetting-free antibody staining procedure, which should reduce the influence of pipetting-associated errors and costs, and enhance the standardization of detection [2].

Beckman Coulter® recently developed the DuraClone® IF Basophil Activation Assay. It utilizes tubes containing a dried five-color antibody panel that is specific for the detection of activated basophils by flow cytometry.

**Fig. 1:**
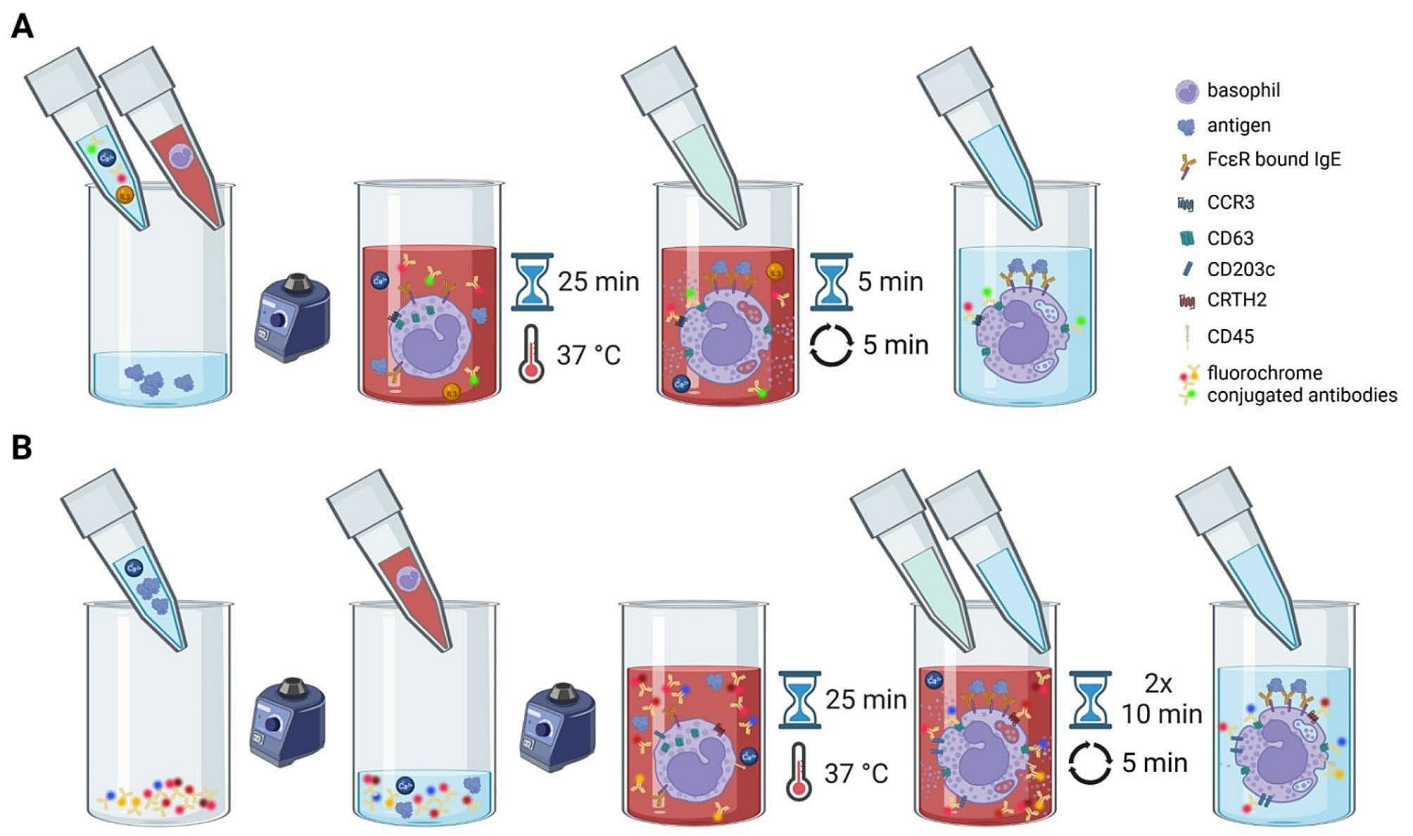
Schematic illustration of BAT protocols (A) BAT 1 and (B) BAT 2 (created with BioRender.com). BAT 1: Antigens, buffer, staining reagents and blood are added to the tube before incubation. BAT 2: Antigens and blood are added to the tube with dried antibodies before incubation

The aim of this study was to assess the performance and suitability of the DuraClone® IF Basophil Activation Assay in comparison with another commercially available and well-established test (FlowCAST®), that utilizes liquid reagents (Fig. 1). This comparison was done within the realm of advanced allergy diagnostics for patients with insect venom allergy.

## Materials and methods

### Patients

Seventeen patients (11 men and 6 women) ranging in age from 28 to 77 years (mean age: 52.9 ± 16.6 years) with an insect venom allergy (positive history of a systemic reaction to a bee, wasp or hornet sting, positive skin test results to bee and/or wasp venom and positive sIgE results to bee and/or wasp venom) were included in the study. More details can be found in Table S1. The study was approved by the Technical University of Munich Ethics Committee [protocol #5478/12], and all participants provided written informed consent. The study was performed in accordance with the Declaration of Helsinki.

### Allergens

Honey bee venom (HBV) and yellow jacket venom (YJV) were commercially available in lyophilized form of 312.5 ng per vial (BAG2-I1CHK, BAG2-I3CHK; Bühlmann Laboratories AG, Schönenbuch, Switzerland). They were then diluted in 250 µl stimulation buffer (containing heparin, Ca^2+^, and IL-3 [2 ng/mL]) from Bühlmann Laboratories for basophil activation test 1 (BAT 1) or in Activation Solution (buffer, containing Ca^2+^) from Beckmann Coulter (Brea, California, USA) for basophil activation test 2 (BAT 2). Three dilutions (1:1, 1:5, 1:25) of this stock solution (1250 ng/ml) were used.

### Basophil activation tests

Basophil activation test 1 (BAT 1): This test was performed as previously described [13], using the FlowCAST® (Bühlmann Laboratories AG, Schönenbuch, Switzerland).

Venous blood was collected in 10 mL EDTA tubes and stored at 4 °C. The anticoagulated blood samples were gently homogenized by inverting them several times. For each patient and allergen, polystyrene tubes were prepared with 50 µl of allergen. Monoclonal anti-FcεRI antibody (anti-FcɛRI mAb) and N-formyl-methionyl-leucyl-phenylalanine (2 mM) were used as positive controls, while stimulation buffer alone was used as a negative control. Subsequently, 100 µL of stimulation buffer containing calcium, heparin and IL-3 (2 ng/mL), 50 µL of blood and 20 µL of staining reagent (anti-CD63-FITC and anti-CCR3-PE mAbs) were added to the antigen dilutions and incubated at 37 °C in a water bath for 25 min. Stimulation was stopped by adding 2 mL of lysis buffer for 5 min at room temperature. After centrifugation for 5 min at 500 x g, the supernatant was decanted and 300 µL of washing buffer was added to each tube (Fig. 1). Each sample was analyzed once.

Cells were analyzed by flow cytometry on a FACSCalibur flow cytometer (Becton-Dickinson Immunocytometry System, Heidelberg, Germany) equipped with lasers at 488 nm and 633 nm. Analysis was performed using the software BD CellQuest Pro. Basophils were identified within the lymphocyte population using anti-CCR3 and the upregulation of the activation marker CD63 was determined by calculating the percentage of CD63^high^ basophils out of the total. The cut-off was set at 10% CD63^high^ cells as recommended by the supplier.

Basophil activation test 2 (BAT 2): The Dura Clone IF Basophil Activation® test from Beckman Coulter, Inc. in Brea, California, USA was used for this test. The Beckman Coulter test kit included two types of test tubes with antibodies conjugated with fluorochromes that were already dried and fixed. The tubes for the positive controls contained the following antibodies: anti-CD45-KrO, -CD3-PC7, -CRTH2-AF647, -CD203c-PE, -CD63-PacBlue and anti-IgE. The tubes for the allergens contained antibodies anti-CD45-KrO, -CD3-PC7, -CRTH2-AF647, -CD203c-PE and -CD63-PacBlue. 50 µl of allergen was used for the allergen tubes and for the positive control tubes 50 µl of activation solution was added. To compare the positive control with the FlowCAST® an additional 50 µl of the monoclonal anti-FcεRI antibody was added to an allergen tube. The tubes were vortexed at high speed for 6 to 8 s. 50 µl of blood was then added. The tubes were gently vortexed for 1 to 2 s and incubated for 25 min in an incubator at 37 °C. 250 µl of OptiLyse C from Beckman Coulter (containing 1.5% formaldehyde) was added. The tubes were immediately vortexed for 1 to 2 s to initiate erythrocyte lysis and then incubated for 10 min at room temperature in the dark. Lysis was stopped by adding 250 µl of PBS (without Ca^2+^/Mg^2+^). The tubes were vortexed for 1 to 2 s and incubated for 10 min at room temperature. 3 ml of PBS was added. The tubes were then centrifuged for 5 min at 500 x g at room temperature. The supernatant was aspirated and the cells were resuspended in 300 µl of PBS (Fig. 1). Each sample was analyzed once.

Cells were analyzed using a FACSCanto™ II flow cytometer (Becton-Dickinson Biosciences GmbH, Heidelberg, Germany) with lasers at 405 nm, 488 nm, and 633 nm. The analysis was performed by using BD FlowJo™ Software (BD Life Sciences).

### Gating strategies

**Fig. 2:**
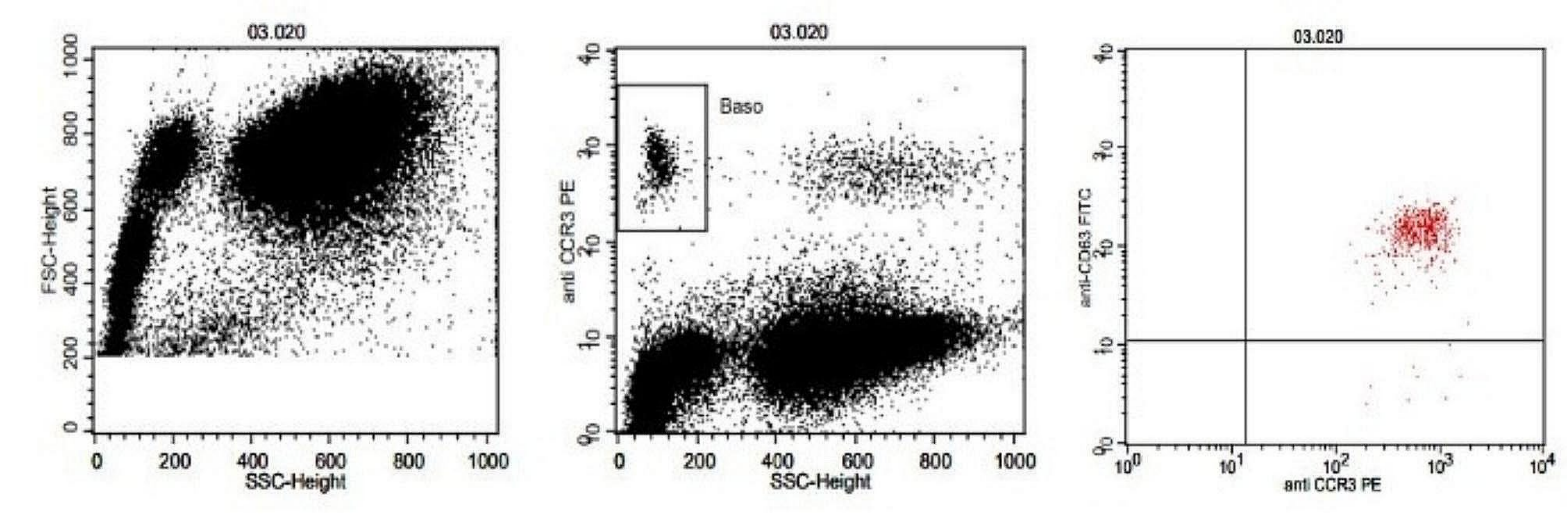
Gating strategy of BAT 1. Example of an analysis with the positive control anti-FcɛRI antibody. A Forward Scatter/Side Scatter plot was created to acquire the whole leukocyte population separated into three discrete populations (lymphocytes, monocytes and granulocytes). Plot 2 was created (CCR3-PE vs. Side Scatter) and basophils were gated as CCR3^pos^ and SSC^low^. Plot 3 was created (CD63-FITC vs. CCR3-PE) to determine stimulated basophils. Activated basophils result in a CD63 positive basophil population (CD63^pos^/CCR3^pos^/SSC^low^) identified in the upper right quadrant

The gating strategy for the basophil activation test 1 was as follows according to the manufacturer´s instructions (Fig. 2):

A dot plot 1 was created (Forward Scatter vs. Side Scatter) to acquire the whole leukocyte population and exclude debris. During sample acquisition, it was ensured that the leukocyte population was separated into three discrete populations (lymphocytes, monocytes and granulocytes) on the FSC/SSC plot. A dot plot 2 was created (CCR3-PE vs. Side Scatter) and basophils were gated as CCR3^pos^ and SSC^low^. Eosinophils, which are also CCR3^pos^, were excluded based on the high SSC. In the next step, a dot plot 3 was created (CD63-FITC vs. CCR3-PE) to determine stimulated basophils. The non-stimulated, resting basophils of the patient background tube were used to set a quadrant gate including CD63 negative basophil cells in the lower right quadrant (CD63^neg^ CCR3^pos^/SSC^low^). Basophils activated by the stimulation of positive controls and specific allergens will result in a CD63 positive basophil population (CD63^pos^/CCR3^pos^/SSC^low^) identified in the upper right quadrant. The readout of the assay is indicated as the ratio of CD63 positive basophils over all basophils (%CD63 activation) as identified in the quadrant gate of the dot plot 3 for any of the stimulation tubes. 500 or more basophilic cells were acquired for any stimulation tube (gated as shown in plot 2).

**Fig. 3:**
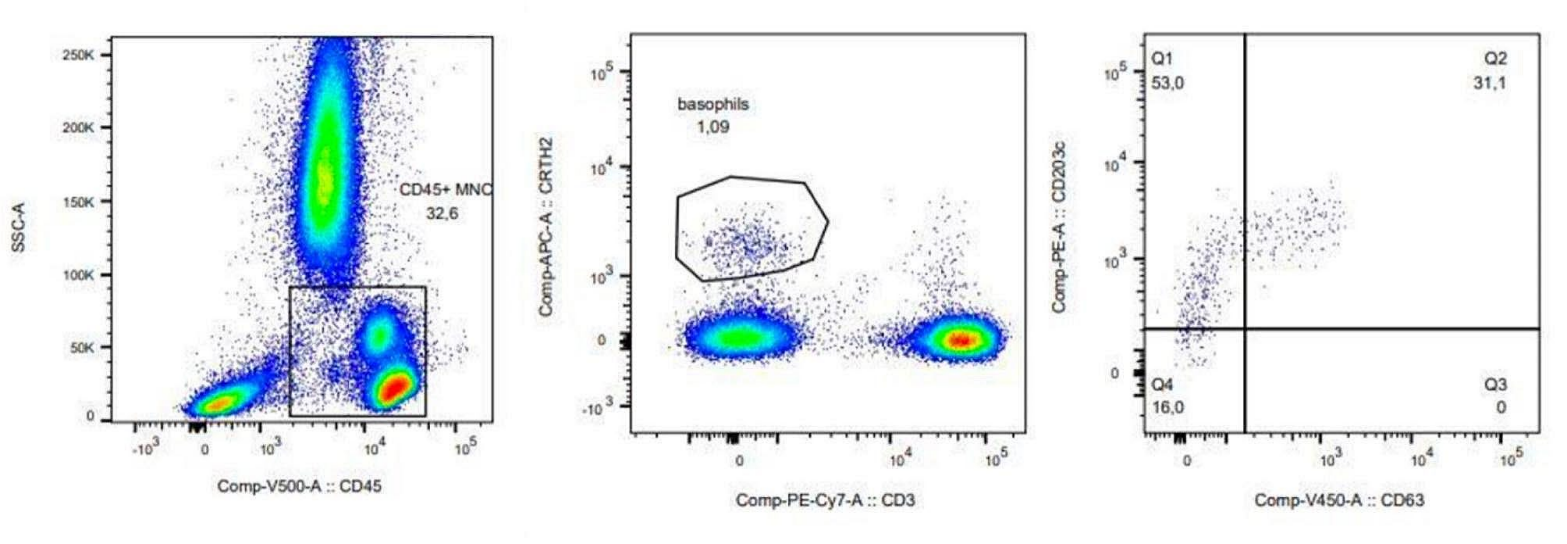
Gating strategy of BAT 2. Example of an analysis with yellow jacket venom (YJV). A CD45-Krome Orange vs. SSC-A dot plot was created. A region was drawn to encompass the CD45^pos^ leukocytes. A plot 2 (CD3-PC7 vs. CD294 (CRTH2)-Alexa Fluor 647) was created and the leukocyte gate was applied to the plot. A plot 3 (CD63-PacBlue vs. CD203c-PE) was created and the CRTH2^pos^ CD3^neg^ cells gate was applied to this plot. A quadrant gate was selected and applied to this plot. Activated basophils result in a CD63^pos^/CD203c^pos^ basophil population in the upper right quadrant

The gating strategy for the basophil activation test 2 was as follows (Fig. 3):

A CD45-Krome Orange vs. SSC-A dot plot was created and the cell gate was applied to this plot. A region was drawn to encompass the CD45^pos^ leukocytes. A CD3-PC7 vs. CD294 (CRTH2)-Alexa Fluor 647 dot plot was created and the leukocyte gate was applied to the plot. A CD63-PacBlue vs. CD203c-PE dot plot was created and the CRTH2^pos^ CD3^neg^ cells gate was applied to this plot. A quadrant gate was selected and applied to this plot. The quadrant lines were adjusted to delineate the CD63^neg^/CD203c^neg^, CD63^neg^/CD203c^pos^, CD63^pos^/CD203c^neg^ and CD63^pos^/CD203c^pos^ cells. The latter are regarded as activated basophils. 500 or more basophilic cells were acquired for any stimulation tube.

### Cut-offs

It was determined that for both BAT 1 and BAT 2 a negative control below 5% (with the exception of P26 who had a negative control of 9.3% in BAT 2) and stimulation controls above 10% must be achieved for a usable result as specified by the manufacturer of BAT 1. The cut-offs of 10% suggested by the manufacturer of BAT 1 were considered for HBV and YJV.

### Statistical analysis

For the graphical comparison of the two tests, we created a Bland-Altman plot. This plot allows us to draw conclusions about the concordance of the two measurement methods. The differences between the pairs of measured values on the Y-axis are plotted against the mean value on the X-axis. Additionally, the mean value of the differences and two limits of agreement (LoA; d ± 1.96 x standard deviation) were plotted as horizontal lines. This provides information about the dispersion of the differences [3].

Since the interpretation of the BAT makes a dichotomous statement (activation above or below the cut-off), we also conducted an analysis using Cohen’s kappa. This measure assesses the agreement of judgments with nominal values, giving insight into interrater reliability.

Cohen’s kappa can range from 0 to 1. A value of 1 indicates complete agreement between the methods. If the match between methods is no better than chance, Cohen’s Kappa is 0 [11]. According to Landis/Koch [12], the interpretation of Cohen´s Kappa values is as follows (value (κ)/extent of agreement): < 0.00/poor; 0.00–0.20/slight; 0.21–0.40/fair; 0.41–0.60/moderate; 0.61–0.80/substantial; 0.81-1.0/(almost) perfect.

The statistical analysis and graphical presentations were conducted using IBM SPSS Statistics 20, Microsoft Office Excel 365 and BioRender software.

## Results

### Exclusion of patients (Fig. 4)

**Fig. 4:**
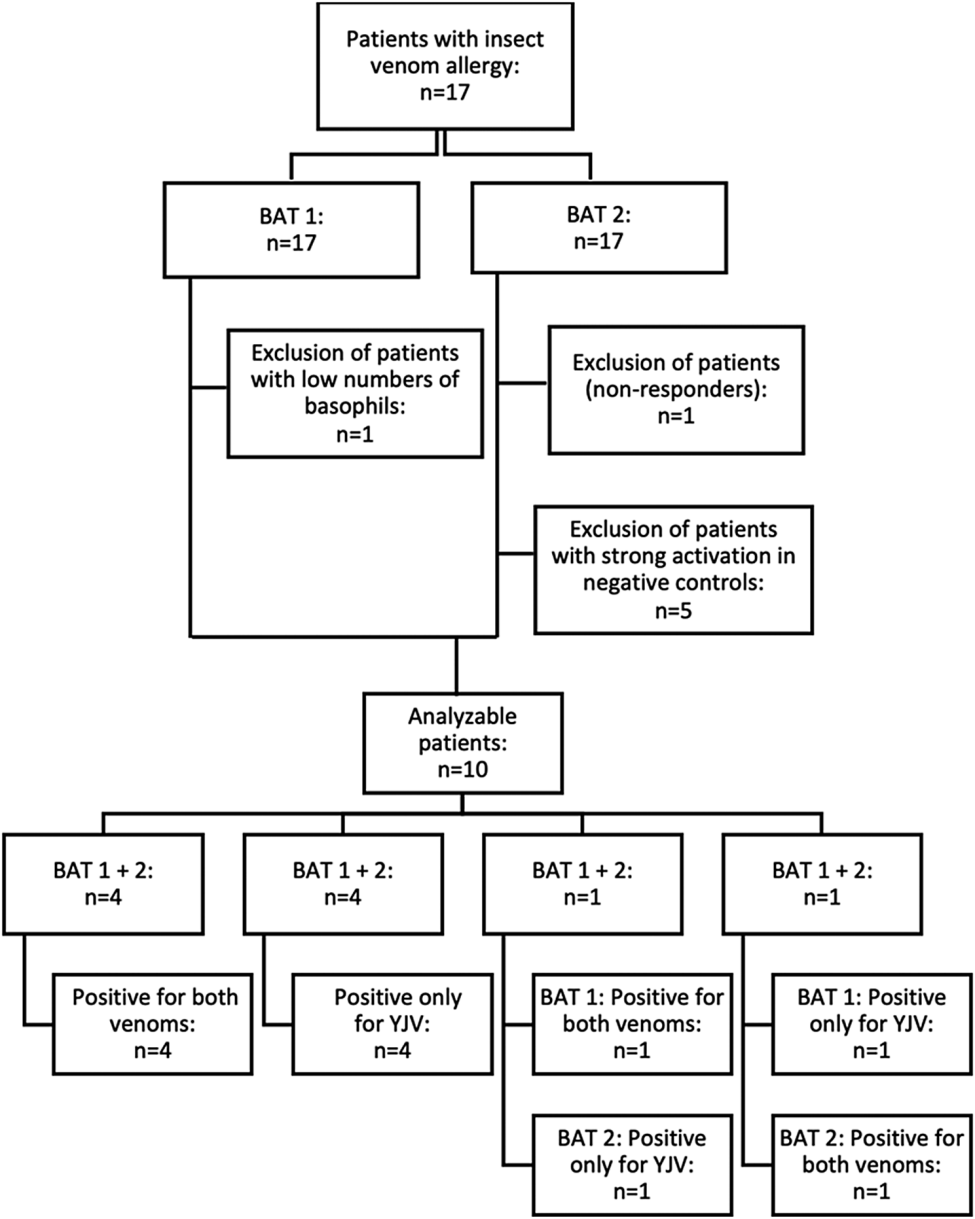
Study design with exclusion criteria of patients and results of the qualitative statements of both tests (BAT 1 and BAT 2). HBV, honey bee venom; YJV, yellow jacket venom

One patient (P29) was withdrawn from the study using BAT 1 because the number of basophils required for analysis was too low. As a result, BAT 2 was also not performed. Another patient (P23) did not show response to the positive controls in BAT 2 above the defined cut-off of > 10% (results: anti-IgE: 2.1%) and was classified as a non-responder.

Five patients (P5, P21, P30, P31 and P33) showed strong activation in the negative controls (mean ± SD: 50.1 ± 5.0%) in BAT 2, while the values in BAT 1 were within the normal range (< 5%). Due to these inconclusive results, these patients were excluded from the quantitative and qualitative analyses.

### Quantitative comparison of results

For the quantitative comparison of the results of both tests, the differences in the respective values for negative controls, dilutions of the allergens (HBV and YJV), and the positive control anti-FcɛRI mAb were analyzed. The mean (± SD) differences for the single samples can be found in Table 1.

**Table 1:** Evaluation of the differences between activation (%CD63) using BAT 1 and BAT 2. HBV, honey bee venom; YJV, yellow jacket venom; 1, concentration 1; 2, concentration 2; 3, concentration 3

Substance	Mean	Maximum	Minimum	SD
**Blank**	-0.2	8.7	-4.4	2.8
**Anti-FcεRI**	-10.1	-0.7	-30.2	9.3
**HBV 1**	0.1	34.5	-28.9	17.8
**HBV 2**	5.4	46.5	-7.8	14.7
**HBV 3**	1.5	13.9	-5.3	5.3
**YJV 1**	-6.2	15.2	-20.3	14.3
**YJV 2**	0.9	22.6	-17.6	11.8
**YJV 3**	4.4	34.9	-2.1	10.3

**Fig. 5:**
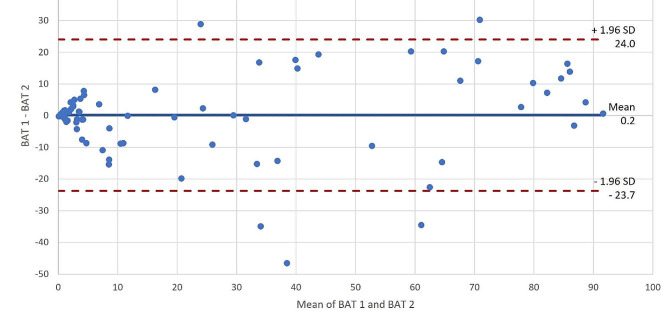
Comparison of BAT 1 and BAT 2 (% activation) in a Bland-Altman Plot (*n* = 84). The Limits of Agreement ranged from 24.0 to -23.7

The overall mean (± SD) difference was 0.2 (± 12.2) %P. Figure 5 shows the comparison of both tests in a Bland-Altman plot. The limit of agreement (LoA) ranged from 24.0 to -23.7, meaning that 95% of the differences to be measured in the future are expected to lie in this interval ([-23.7; 24.0]). This corresponds to a smallest detectable change (SDC) of 47.7. For values below the cut-offs of 10%, the scattering was substantially lower than for higher mean values.

### Qualitative statements of the two tests (Fig. 4)

In interpreting the results of the BAT, the qualitative statement is dichotomous, indicating activation above the cut-off or not. The qualitative evaluation examined whether the two test kits produced similar results based on the 10% cut-off values defined for the allergens. In eight out of the ten patients evaluated (P15, P22, P24, P25, P27, P28, P32, P34), both tests yielded the same activation status for HBV or YJV. Specifically, in four patients (P15, P22, P27, P34), no activation above the cut-off for HBV was observed, while YJV did lead to basophil activation. Four patients (P24, P25, P28, P32) showed activation for both venoms.

In two out of ten cases, the qualitative results of the two test kits were different. For instance, in P6, activation was detected for both venoms at the highest concentration with BAT 1, while with BAT 2 only the YJV at the highest concentration showed activation above the cut-off. Conversely, in BAT 2, patient P26 exhibited activation for both venoms (all concentrations of HBV and YJV) whereas in BAT 1 activation was only observed for YJV.

Cohen’s kappa was calculated to evaluate judgmental agreement by comparing the results for each of the three concentrations of HBV and YJV. The potential nominal statements were either “activation below the cut-off” or “activation above the cut-off”. In 26 out of the 60 cases, both BAT 1 and BAT 2 identified activation below the cut-off, while in 27 cases, they identified activation above the cut-off. The relative agreement was 88.3% with a probability of chance agreement of 49.7%. resulting in a Cohen’s kappa of 0.77.

In five patients (P22, P24, P25, P27, P32), the results of both tests were consistent with the previously diagnosed allergy and sensitization profile. For two patients (P15, P34), the results for HBV were negative in both tests, despite the presence of bee venom sensitization and clinically relevant allergy. In P28 both tests showed positive results for HBV, which aligned with the specific IgE determination but not with the diagnosed allergy (YJV allergy only). For the two patients (P6, P26) with qualitatively different results, BAT 1 supported the previous diagnosis (P6: HBV and YJV allergy; P26: exclusive YJV allergy). However, BAT 2 showed a negative result in P6, who had sIgE and a clinically relevant allergy to HBV. In P26, BAT 2 results were positive for HBV and YJV, consistent with the sensitization profile when specific IgE was determined, but not with the diagnosed YJV allergy (Table S1)).

### Comparison of positive controls anti-FcɛRI mAb (BAT 1) and anti-IgE (BAT 2)

**Fig. 6:**
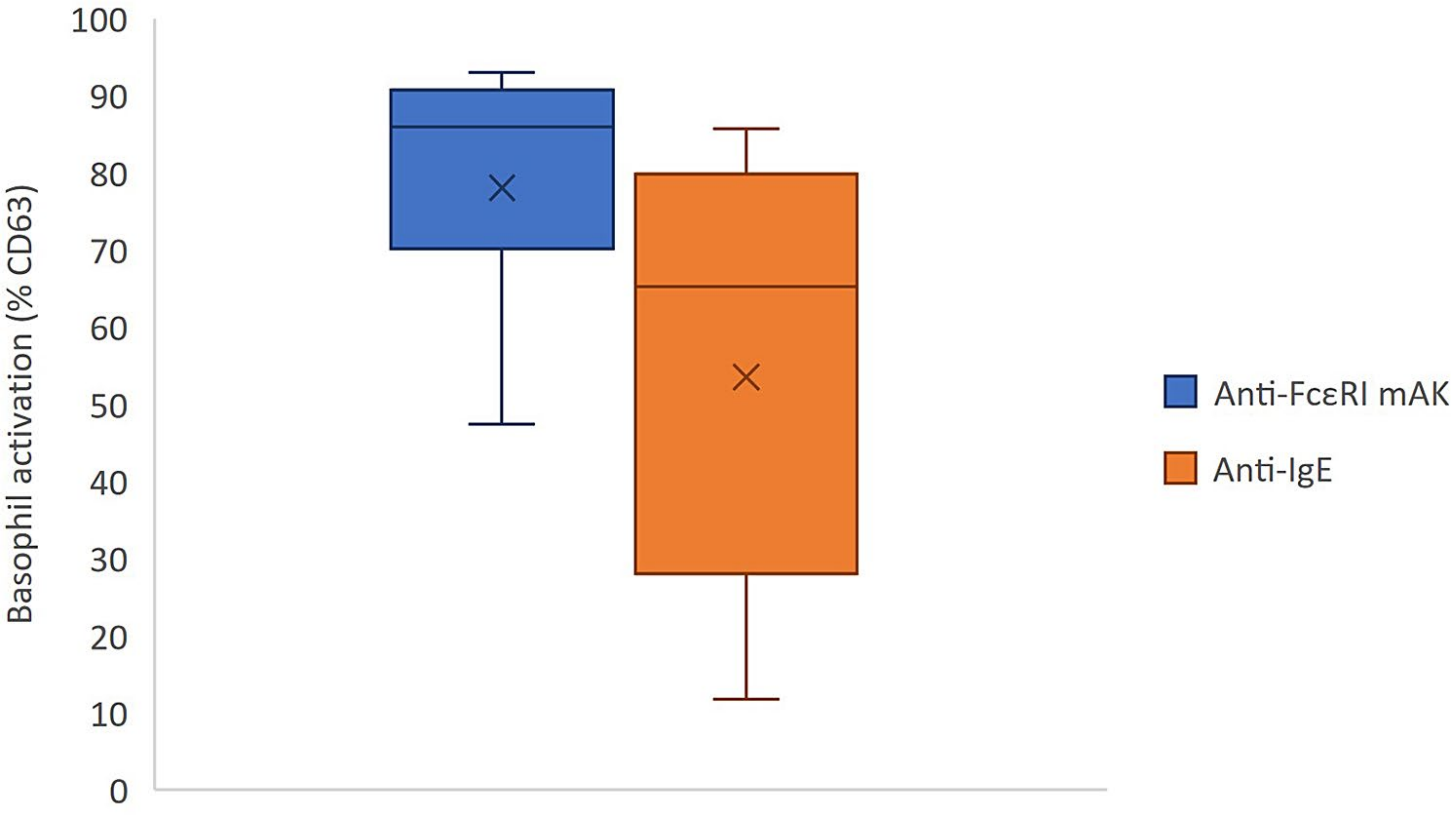
Distribution of the measured basophil activation by the positive controls anti-FcɛRI mAb and anti-IgE with median (line), mean (X), minimum, maximum and quartiles

Both positive controls resulted in sufficiently high activation above the 10% cut-off in the 10 patients studied. Basophil activation by anti-FcɛRI mAb in BAT 1 was significantly higher than by anti-IgE in BAT 2 (mean activation: 78.1% (± 16.6%) vs. 53.6% (± 28.4%); *p* < 0.01). Values for anti-IgE showed a wider range from 11.8 to 85.7% (Fig. 6).

### Comparison of execution times

Sixteen patients were included in the comparison of execution times for the two test kits.

On average, the execution time for BAT 1 was 1 h, 8 min, and 13 s (± 9 min, 58 s) while for BAT 2 it was 1 h, 30 min, and 36 s (± 10 min, 54 s). The mean difference per patient examined was 22 min and 13 s (± 5 min, 38 s). Overall, BAT 2 required approximately 1.3 times the execution time of BAT 1 due to additional procedures (double addition of PBS buffer, third incubation step).

### Cost comparison

A cost comparison between BAT 1 and BAT 2 was based on the test capacity of each kit. BAT 1 can perform 100 tests per kit without additional reagent costs, while BAT 2 allows 25 tests per kit and requires extra reagents. BAT 2 was found to be more expensive per test than BAT 1.

## Discussion

The comparison between BAT 1 and 2 revealed overall sufficient agreement between the tests, indicating that the newer test with fixed antibodies is suitable for testing insect venom allergens in terms of results. However, there are some aspects that demonstrate the advantages of the established test.

One important factor to consider is the use of different flow cytometers, software analyses and gating strategies for the two tests. BAT 1 utilized a flow cytometer used in routine diagnostics and research, but only had 2 lasers. In contrast, BAT 2 required a three-laser configuration, necessitating the use of a different flow cytometer. The gating strategies were adjusted according to the provided BAT protocols and could not be changed. However, the non-specific activation of negative controls mentioned below is unlikely to be attributed to these factors.

In BAT 2, five out of 17 tests resulted in high non-specific activation with values of the negative controls ranging from 41.7 to 60.5% (expected values < 5%). These results did not allow for a meaningful evaluation of the tests with allergens. In BAT 1 and in a manual test, where the antibodies of BAT 2 were used as liquid antibody reagents (data not shown), expected values (< 5%) of the negative controls were observed. The cause of the high blank values remains unclear but seems to be associated with the special technique of BAT 2 with the tubes containing dried antibody reagents. In BAT 1, an additional buffer solution of 100 µL was added before the blood sample, while in BAT 2 no additional buffers were used. It can be speculated that this difference may affect the stability and integrity of basophils, resulting in varying levels of non-specific activation. The individual sensitivity of the cells could contribute to the non-specific activation observed in some patients.

IgE signaling-dependent positive controls resulted in activations above the cut-off in both tests leading to usable results. However, the anti-Fc𝜀RI antibody used in BAT 1 showed higher activation and less scatter than the anti-IgE used in BAT 2. This difference should be considered when calculating the CD63 ratio, which is the ratio of allergen-induced activation to the IgE signaling-dependent positive control [13, 15, 17]. Additionally, this discrepancy led to the exclusion of one patient in the overall evaluation, as he was classified as a non-responder in BAT 2 with a value of 2.1% (anti-IgE), but would have been a responder in BAT 1 using anti-Fc𝜀RI (22.2%). The use of anti-FcɛRI mAb as a positive control in BAT 2 led to significant activation, demonstrating its effectiveness also in BAT 2 (Table 1).

The levels of the measured activations were compared in a Bland-Altman plot, with the interval of the LoA being [-23.7; 24.0]. The scatter for values around the cut-off was lower than for higher values indicating activation. The interpretation of the LoA is based on the clinical question, with an interval of [-10; 10] %P being conceivable in the present study. Under these conditions, one would assume a clinically insufficient agreement.

When interpreting the BAT, categorical statements with nominal expressions (“activation below/above the cut-off”) are often more important than the percentage activation they are derived from. In the study of the allergens HBV and YJV, the measure of judgment agreement was determined using Cohen’s Kappa and found to be k = 0.77. According to the categorization proposed by Landis and Koch, this indicates a substantial agreement (cut-off values: 0.61–0.80).

In detail, the results were consistent in eight out of 10 patients while two patients showed different results. For instance, in P6 with a positive history and sensitization to HBV and YJV, the result corresponded better with BAT 1 (HBV and YJV positive) than with BAT 2 (HBV negative, YJV positive). In P26, a negative intradermal test and lack of IgE to recombinant bee venom allergens indicated no HBV sensitization aligning more with the results of BAT 1 (HBV negative, YJV positive) than with BAT 2 (HVB and YJV positive). Two patients with a confirmed HBV allergy showed no activation by HBV in either test, indicating false-negative results in both tests. This could be due to low concentrations of single components in the total venom extract used.

The analysis revealed that BAT 2 took 22 min and 23 s longer than BAT 1 (1.3 times longer) mainly due to a longer incubation time of 45 min in BAT 2 compared to 30 min in BAT 1. The wash step described as optional in BAT 2 could not be omitted due to longer FACS analysis time and interference from cell debris in preliminary tests. When comparing costs, the cost of one test was higher for BAT 2 than for BAT 1.

BAT 2 only contains five positive controls, limiting the number of patients that can be analyzed to a maximum of five per kit. In contrast, the positive control in BAT 1 was included as a liquid reagent making it generally more flexible in experimental setups.

The technique of using dried antibodies for flow cytometric stains was also applied in hemato-oncology and compared with established methods. While the method allowed for accurate detection of minimal residual disease in multiple myeloma and chronic lymphocytic leukemia, discrepancies were observed [2, 19].

In conclusion, the technique of BAT 2 represents an interesting innovation in commercially available BATs but appears less suitable for routine diagnosis of insect venom allergy compared to established tests due to non-specific basophil activation, longer execution time, and higher costs.

### Limitations

The current study has certain limitations that should be considered.

The sample size of the study included 17 patients with insect venom allergy. Although the inclusion criteria were carefully applied, the limited sample size may restrict the broader applicability of the findings to a larger population. Further studies with a larger sample size including more patients are needed to validate and extend the current findings.

Another limitation of this study is the high levels of non-specific activation in negative controls in 5 out of 17 tests using BAT 2. This non-specific activation may interfere with allergen-induced activation making it difficult to interpret the results obtained with BAT 2. The underlying factors should be investigated and strategies for improvement should be developed.

Additionally, this study primarily focused on insect venom allergies, which may limit the generalizability of the findings to other allergens. Future studies including a broader range of allergens could enhance understanding of basophil activation testing across different conditions, improving clinical applicability.

### Electronic supplementary material

Below is the link to the electronic supplementary material.

Supplementary Material 1

## Data Availability

No datasets were generated or analysed during the current study.
