# Supplementary material for: Basophil activation in insect venom allergy: comparison of an established test using liquid reagents with a test using 5-color tubes with dried antibody reagents
Source: BMC Immunol. 2024 Apr 27;25:23. doi: 10.1186/s12865-024-00616-0 (PMC11055254; doi:10.1186/s12865-024-00616-0)
Supplement: Supplementary file 1 — Supplementary Material 1 [file 12865_2024_616_MOESM1_ESM.doc]

Supplementary Material

Table S1: Characteristics of patients

| **Patient-Nr.** | **1** | **2** | **3** | **4** | **5** | **6** | **7** | **8** | **9** | **10** | **11** | **12** | **13** | **14** | **15** | **16** | **17** |
| --- | --- | --- | --- | --- | --- | --- | --- | --- | --- | --- | --- | --- | --- | --- | --- | --- | --- |
| **Patient-ID** | P5 | P6 | P15 | P21 | P22 | P23 | P24 | P25 | P26 | P27 | P28 | P29 | P30 | P31 | P32 | P33 | P34 |
|  |  |  |  |  |  |  |  |  |  |  |  |  |  |  |  |  |  |
| **Allergy diagnosis** | B and W | B, W and H | B and W | B | W | B | B, W and H | B and W | W and H | W | W and H | B | W | W | B and W | B | B and W |
| **Anaphylaxis grade** | II | III | II | I | II | II | I | II | II | IV | II | III | II | II | II | II | I |
| **i.c. Bee venom**  **(µg/ml)** | pos at 0.0001 | neg | pos at 0.1 | pos at 0.0001 | neg | pos at 0.001 | pos at 0.001 | pos at 0.001 | neg | neg | pos at 0.001 | pos at 0.01 | pos at 0.01 | neg | pos at 0.0001 | pos at 0.001 | neg |
| **i.c. Wasp venom**  **(µg/ml)** | pos at 0.001 | pos at 0.01 | pos at 0.1 | neg | pos at 0.0001 | neg | pos at 0.1 | pos at 0.001 | pos at 0.01 | pos at 0.1 | pos at 0.001 | pos at 0.001 | pos at 0.01 | pos at 0.0001 | pos at 0.01 | pos at 0.1 | pos at 0.0001 |
| **Further findings** |  | Masto-cytosis |  |  |  |  |  | RCA saiso-nalis | RCA saiso-nalis, OAS | Masto-cytosis |  | Masto-cytosis |  |  |  |  |  |
| **Total-IgE (IU/ml)** | 364 | 14.4 | 20.4 | 127 | 89.3 | 49.4 | 284 | 207 | 234 | 117 | 127 | 186 | 22.6 | 108 | 6.97 | 54.4 | 196 |
| **sIgE bee venom** Class | 3 | 4 | 2 | 6 | 2 | 4 | 5 | 3 | 3 | 0 | 3 | 2 | 2 | 3 | 1 | 2 | 3 |
| **sIgE rApi m 1**  Class | 1 | 3 | 2 | 4 | 0 | 4 | 4 | 2 | 0 | 0 | 1 | 2 | 2 | 0 | 0 | 2 | 0 |
| **sIgE rApi m2** Class | 3 | 2 | 3 | 0 | 0 | 0 | 0 | 0 | 0 | 0 | 3 | 0 | 0 | 1 | 0 | 0 | 0 |
| **sIgE rApi m3** Class | 0 | 2 | 1 | 3 | 0 | 2 | 3 | 2 | 0 | 0 | 0 | 0 | 0 | 0 | 0 | 0 | 0 |
| **sIgE rApi m5**  Class | 1 | 0 | 0 | 3 | 0 | 0 | 2 | 0 | 0 | 0 | 2 | 0 | 0 | 0 | 0 | 1 | 0 |
| **sIgE rApi m10**  Class | 0 | 3 | 0 | 2 | 0 | 3 | 3 | 2 | 0 | 0 | 2 | 0 | 0 | 1 | 0 | 0 | 2 |
| **sIgE wasp venom**  Class | 3 | 2 | 3 | 2 | 5 | 1 | 3 | 2 | 3 | 2 | 4 | 2 | 2 | 5 | 2 | 2 | 3 |
| **sIgE r Ves v1**  Class | 1 | 0 | 3 | 2 | 0 | 0 | 0 | 0 | 2 | 1 | 2 | 0 | 0 | 3 | 0 | 0 | 0 |
| **sIgE rVes v 5** Class | 3 | 2 | 0 | 0 | 5 | 1 | 2 | 2 | 3 | 2 | 3 | 2 | 0 | 4 | 1 | 2 | 3 |
| **sIgE hornet**  Class |  | 1 |  |  |  |  | 3 |  |  |  | 3 |  |  |  |  |  |  |
| **CCD, MUXF3**  Class | 2 | 0 | 0 | 0 | 2 | 0 | 3 | 0 | 2 | 0 | 2 | 0 | 0 | 3 | 0 | 0 | 0 |
| **Tryptase (µg /l)** | 5.08 | 36.6 | 6.58 | 5.51 | 4.0 | 4.76 | 5.87 | 7.61 | 3.09 | 20.4 | 3.56 | 26.7 | 5.69 | 2.2 | 5.09 | 3.72 | 14.3 |

B, bee; H, hornet; neg, negative; OAS, oral allergy syndrome; pos, positive; RCA, rhinoconjunctivitis allergica; W, wasp
